# Transmission Control Protocol (TCP)-Based Delay Tolerant Networking for Space-Vehicle Communications in Cislunar Domain: An Experimental Approach

**DOI:** 10.3390/s25041136

**Published:** 2025-02-13

**Authors:** Ding Wang, Ruhai Wang

**Affiliations:** 1 School of Mathematics and Computer Science, Northwest Minzu University, Lanzhou 730030, China; 185142481@xbmu.edu.cn; 2 Phillip M. Drayer Department of Electrical and Computer Engineering, Lamar University, Beaumont, TX 77710, USA

**Keywords:** space vehicular networks, space vehicle communications, internet of vehicles, vehicular ad hoc networks, TCP, DTN

## Abstract

The integrated heterogeneous 7G/8G networks may face multiple challenges for reliable data delivery such as link disruption, intermittent link availability, long latency and a highly lossy channel. Delay tolerant networking (DTN) was proposed as a highly reliable networking technology for space networks that will be part of future 7G/8G networks. In this paper, an experimental evaluation of transmission control protocol (TCP)-based DTN (i.e., running TCP at the transport layer of DTN) for space-vehicle communications in the cislunar domain is presented. The impact of link disruption is also considered. The evaluation was conducted using the DTN protocol suites over a realistic experimental testbed. The study results show that TCP-based DTN works effectively for space-vehicle communications in cislunar domain in the presence of a link disruption event. However, a roughly exponential goodput decrease is observed with a linear increase in link delay from 1250 ms to 5 s.

## 1. Introduction

Space networking is characterized by long delays, random link disruptions and a high channel error rate. A lot of effort has been made in the past two decades in studying the satellite/space networks [1]. Delay tolerant networking (DTN) [2] was proposed as a highly reliable networking technology for space networks. Designed as DTN’s baseline protocol, bundle protocol (BP) [3] was expected to provide data delivery over a channel accompanied by long delay and intermittent connectivity (i.e., having frequent link outage). BP is designed to build a networking “overlay” over heterogeneous networks for which various data transport protocols are operable, including the terrestrial TCP [4] and UDP [5].

It is expected that future 7G/8G networks will have seamless global and interplanetary coverage, a significantly fast data rate, low latency, an enormously large network capacity and highly intelligent features. It is very hard to compare how future 7G/8G networks will exactly differ from the current networks because 5G/6G networks are still under study. However, 7G/8G networks are expected to incorporate the TCP/IP-based terrestrial Internet and space-based networks, including the cislunar and deep-space networks. Space communication networks will be integrated as a component of future 7G/8G networks for the expected large coverage. In addition, the Internet of vehicles (IoVs) [6], vehicular ad hoc networks (VANETs) [7], edge computing [8] and deep reinforcement learning [9] are also expected to be incorporated for future 7G/8G networks. This implies that space networks will eventually integrate with the IoV and VANETs with both terrestrial and space vehicles incorporated. The integrated heterogeneous 7G/8G networks may face multiple challenges for reliable data delivery such as intermittent link availability, link disruption, long latency and a highly lossy channel.

Data link interruption is an inevitable event over space communication channels. Because of spacecraft movement, planetary rotations, cosmic radiation and interstellar dust, the link disruption may occur randomly or non-randomly with varying durations. Link disruption severely degrades the performance in the deep-space domain because the link for data transfer is unavailable during disruption. The unavoidable long interplanetary propagation delay aggravates the transmission efficiency. The baseline transmission technology for the reliable data delivery of BP mainly includes the store-and-forward method. The store-and-forward service of BP enables DTN to suspend and resume data transfer when links are disrupted. Working together with the nonvolatile memory of a data node, this mechanism ensures that data units of BP, termed bundles [10], are properly stored in case of data link unavailability during data transfer.

Multiple countries have been working on a Moon-landing mission. Effective networking technology is urgently needed for cislunar communications. NASA and several space agencies have been working on implementing space networking (including cislunar and deep-space communication networks) using effective networking architecture and protocols including the TCP-based reliable data transport protocol.

At present, DTN has been considered as the only candidate protocol that has the capability to combat the link outage and link delay in space [11]. However, very few studies have been conducted for a performance evaluation of TCP-based DTN operating under a BP overlay (i.e., running TCP at the transport layer) [12] for cislunar communications, especially using an experimental approach. The main research question is how effective TCP-based DTN works for space-vehicle communications in the cislunar domain in the presence of a link disruption event.

In this study, an experimental evaluation of TCP-based DTN [13] for space-vehicle communications in cislunar domain is presented TCP/IP configured to run underneath BP. The impact of link disruption is also considered. A comparison between TCP-based DTN [12] and another DTN protocol suite running the recently developed Licklider transmission protocol (LTP) [14] at the transport layer (i.e., BP/LTP) is also presented. The evaluation was carried out by sending a data file over a realistic experimental network testbed. TCP-based DTN works effectively for space-vehicle communications in cislunar domain in the presence of a link disruption event (up to 120 s in the experiment). As the main contribution of this study, the experimental results and findings presented in this paper are anticipated to be helpful in understanding the data transmission effectiveness of TCP-based DTN for space-vehicle communications in cislunar domain and, therefore, helpful in better planning and design of cislunar exploration missions.

The DTN protocol stack Is briefly discussed In Section 2, with the previous work related to space DTN also reviewed. The experimental results are presented in Section 3, with the experimental setup briefly introduced at the beginning. The drawn conclusions are provided in Section 4.

## 2. DTN Protocol Stack and Related Work

**Figure 1:**
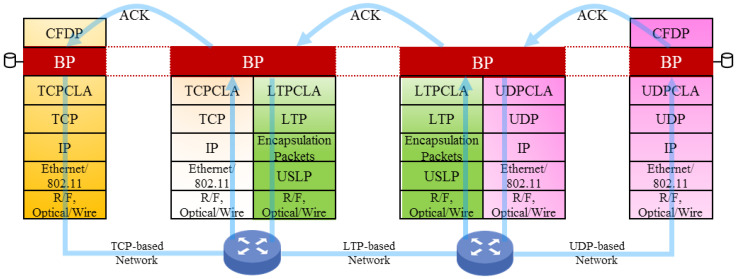
An example of BP-based DTN protocol stack with various protocols running at transport layer.

An example of the DTN protocol stack is shown in Figure 1, illustrating that BP sustains an “overlay” operable over heterogeneous networks. As illustrated, BP utilizes the services of a selected transport-layer protocol via the intermediate “convergence layer adapter” (CLA) [10]. To make BP operable over the TCP-based and UDP-based networks, TCP CLA (i.e., TCPCL) [12] and UDP CLA (i.e., UDPCL) [15] are developed as the intermediate protocol. To facilitate the operation of BP over the new LTP, LTPCL [14] is developed.

As indicated by the names and shown in Figure 1, BP/TCPCL/TCP working with TCP leads to TCP-based DTN operation. In comparison, BP/UDPCL/UDP and BP/LTPCL/LTP provide the UDP-based service and LTP-based service in DTN, respectively. However, all three heterogeneous networks operate under the BP overlay. Readers are suggested to refer to [10] for a discussion related to DTN operation infrastructure and the associated CLAs.

LTP was developed as the primary transport protocol to be utilized in the space domain. LTP targets reliable data transmission over challenging space channel characterized by long link latency and inconsistent link availability. The design and operation of LTP have been extensively described in the lectures [14]; therefore, they are not discussed in detail here.

Some efforts were made in developing DTN technologies for space DTN and its application in cislunar and deep-space domains [16]. While some of them investigated LTP [17], the rest focused on the operation and analysis of BP [18]. A method was proposed in estimating the reliable data delivery time of BP in [19]. In [20], a new model was presented for BP to estimate the optimal length of the retransmission time out (RTO) timer. A few studies were performed to analyze the impact of channel outage on the performance of BP [21]. In [22], a hybrid retransmission mechanism was proposed for BP with an analytical model built to understand the impact of transmission overhead in deep-space communications. Some studies were recently conducted for the performance evaluation of DTN for data delivery from space stations to Earth ground stations in both experimental and analytical manners [23]. Most of these studies focused on the analytical modeling of DTN’s BP and LTP protocols.

The current terrestrial Internet has been dominated by the TCP/IP protocols. It Is expected that a large number of space data nodes will be deployed, and a large number of data links will be available in future space networks. In this case, the effect of frequent link disruption and long delay will not exist. Therefore, it is more likely that the future 7G/8G network will adopt the TCP type of protocol or its extension for reliable transmission. However, none of the aforementioned relative work studies the TCP-based DTN transmission operating under BP. In other words, the findings of these studies are likely inapplicable to the future 7G/8G network. In comparison, this article presents a solid analysis of the TCP-based DTN transmission operating under BP in an experimental manner (especially at data segments level) when applied to cislunar communications. The experimental results presented in this paper are expected to be helpful in understanding the effectiveness and performance of TCP-based DTN for space-vehicle communications.

## 3. Experimental Setup and Study Results

### 3.1. Experimental Setup and Configurations

The experiment presented in this study was conducted by sending a text file running the selected protocol suites over a realistic PC-based network infrastructure, which was the Space Communication and Networking Testbed (SCNT) [16]. The SCNT was designed to emulate the relay type of IPN communication scenarios common in deep-space communications. The performance of the testbed was verified in the previous work [16]. The details of the design and operation of the SCNT were described in the previous studies; therefore, they are not presented here.

In Table 1, the experimental configurations are listed. The factors and their settings configured for the experiment are straightforward and easy to follow. With respect to the protocol implementation and configuration, the file transfer is conducted using a protocol stack of BP/TCP/IP that was adopted from DTN-2. To compare with BP/LTP/UDP/IP, a limited number of experiment runs were also performed with the BP/LTP/UDP/IP protocol stack. Both protocol stacks were adopted from the DTN-2 implementations [13].

Considering that the one-way light time in cislunar communication is generally in a range of 1.25~5 s [24], nine different cislunar link delays in this range were configured for the file transfer experiment. The Point-to-Point Protocol (PPP) [25] was configured at the data link layer, and its data rate is 115,200 bit/s. With this selection, a point-to-point data link was built from the Moon orbiter to the Earth for file transfer, and another one was built over the reverse channel. The channel quality of the experiment was represented by channel BER. It is well known that the classical Additive White Gaussian Noise (AWGN) channel is recognized as an accurate model for the deep-space communication channel by the community [26].

Therefore, the channel BERs in the experiments are generated following the AWGN statistical model. Numerically, three BERs, 0, 10^−6^ and 10^−5^, are configured to represent different channel qualities for the experiment. The selected numerical values of the link delay, channel rates and channel quality in Table 1 are among the widely anticipated channel conditions over deep-space communication channel according to [27].

The experiment was configured with and without the impact of link disruption taken into consideration. The experiment involving link disruption is designed to evaluate the effectiveness of DTN operation in the store-and-forward method and its capability to cope with link-outage events. For the experiment with link disruption, two different disruption events were configured, and their lengths are 60 s (1 min) and 120 s (2 min). The data traffic during the file transfer was dumped using tcpdump over the interfaces of the nodes for performance analysis. The performance analysis toolkits such as Tcptrace [28] and Xplot [29] were used to support the evaluation.

### 3.2. Experimental Results

The experiment results without link disruptions involved are presented first. Then, the experimental results with link disruptions of 60 s and 120 s are presented. For the sake of simplicity, the data-sending node, the data-relay node and the data-receiving node involved for data transmission are simply represented by TX, MX and RX.

#### 3.2.1. Experimental Results Without Link Disruption Event

**Figure 2:**
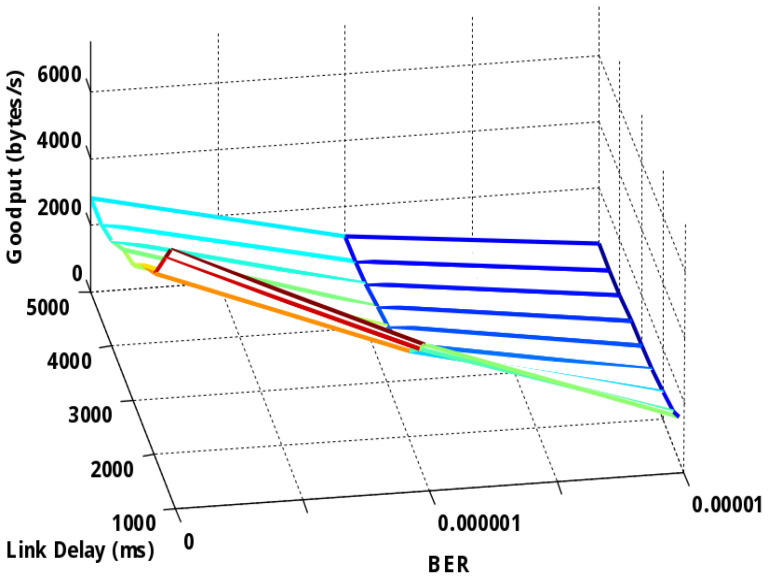
Goodput of TCP-based DTN with respect to the variations of link delay and channel quality (BER).

Figure 2 shows a goodput comparison of the file transfer for TCP-based DTN with respect to the variations of link delay and channel quality (i.e., channel BER). An overall observation is that, for each configured channel quality, all the TCP-based DTN transmissions show goodput degradation with an increase in link delay. Specifically, for a given channel quality, even with a slow additive increase in link delay, the transmissions show an exponential goodput decrease. In comparison, the goodput decreasing rate at a low channel BER is much higher than that at a high channel BER. This is obvious if the goodput is compared between the BERs of 0 and 10^−5^—the variation of the delay at BER = 0 has significant impact on the goodput, while it has only a negligible impact with BER = 10^−5^.

This is especially obvious for the transmissions for which the link delay is short (i.e., from 1250 ms to 2000 ms). This is because at BER = 0, the goodput degradation is dominated by the increase in link delay.

In comparison, with a BER of 10^−5^, the goodput is also severely affected by a high error rate. This is because with an increase in channel error rate, more TCP segments are corrupted. To ensure successful file delivery, these corrupted data have to be retransmitted. The retransmission of more segments leads to an increase in the file delivery time. The longer file delivery time degrades the goodput performance, which is a reflection of the transmission efficiency.

**Figure 3:**
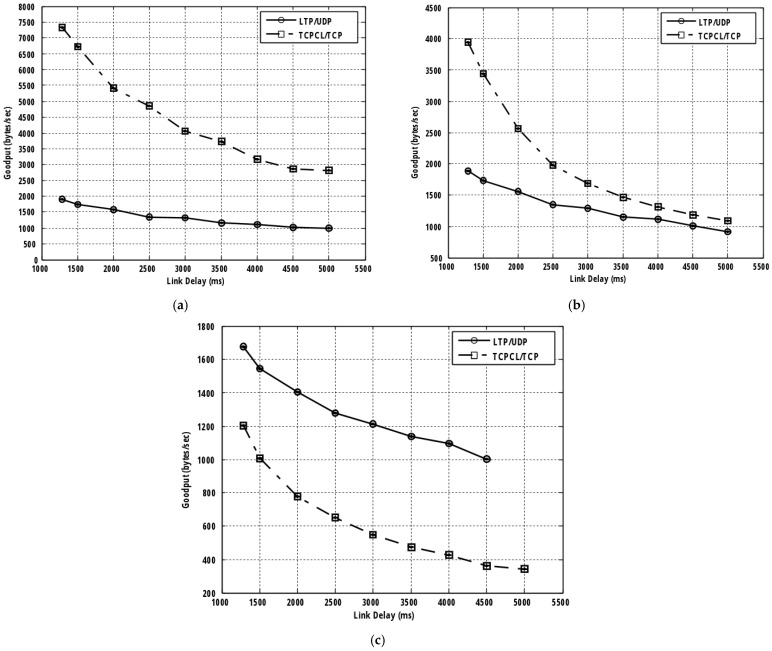
Goodput comparison between TCP-based DTN and BP/LTP/UDP with respect to the variations of link delay and channel quality. (**a**) BER = 0. (**b**) BER = 10^−6^. (**c**) BER = 10^−5^.

In Figure 3, a goodput comparison between TCP-based DTN and LTP-based DTN is presented at each of the three channel BERs. It is obvious that as the BER is configured to be either 0 or 10^−6^, TCPCL/TCP transmission shows goodput advantage over LTP/UDP. The advantage is substantial when the delay is short. For the BER increase from 0 to 10^−6^, the goodput advantage of TCPCP/TCP over LTP/UDP becomes smaller for the reason discussed earlier.

According to the numerical data provided in Table 2, the performance advantage of TCP-based DTN at a BER of 0 is 5425.36 (=7336.36−1911) byte/s and 1825.95 (=2814.95−989) byte/s at the delay of 1280 ms and 5000 ms, respectively. In comparison, at a BER of 10^−6^, the advantages under the same transmission conditions are only 2057.8 (=3940.8−1883) byte/s and 179.4 (=1090.4−911) byte/s. We also see from Figure 3 that at both BERs of 0 and 10^−6^, the goodput advantage of TCP-based DTN becomes smaller with the increase in link delay. With BER = 10^−6^, when the link delay grows as long as 4000 ms or longer, the goodput of the two protocols tends to merge. However, surprisingly, the situation becomes opposite at the BER of 10^−5^.

It is observed that over a noisy or lossy channel having a higher BER, the goodput advantage of TCP-based transmission diminishes, and LTP/UDP shows a goodput advantage at all the experimented link delays. We also see that the performance advantage of LTP/UDP becomes larger when the link delay increases. As seen from Table 2, the advantage is 473.6 (=1675−1201.4) byte/s at a delay of 1280 ms and is 640.3 (=1001−360.7) byte/s at a delay of 4500 ms. This implies that in cislunar communications, TCP-based DTN is more favorable than LTP/UDP over an ideal channel or less lossy channel having a low error rate (i.e., a BER of 10^−6^ or lower). In contrast, LTP/UDP is preferred over TCPCL/TCP over a noisy channel with a high BER, 10^−5^, especially in the presence of a very long propagation delay of 2000 ms or longer. In this case, the performance advantage of LTP/UDP is even more than 630 bytes/s.

The operational differences of two protocols explains the observed performance differences at low and high BERs. At a low BER, a very limited number of retransmissions (because of a very few corruptions) make no difference in the performance of the two protocols with respect to the deferred retransmissions using the selective-acknowledgment reception report of LTP and the interactive, acknowledgment-based immediate retransmission of TCP. So, two protocols have almost no difference in their file transfer time and goodput performance. However, compared to TCPCP/TCP, the interaction between LTP and UDP causes some more processing time for LTP/UDP transmissions in soliciting the selective acknowledgment receipt report for the purpose of retransmission, leading to additional file transfer time and goodput degradation. The interaction time between TCPCL and TCP is much shorter as it is only for the store-and-forward operation, which is also performed for LTP/UDP transmissions.

The performance advantage of TCP-based DTN becomes smaller with the increase in link delays. This can be easily explained. Compared to other transmission factors, a long link delay dominates the data delivery time and, therefore, the goodput for both protocol stacks. With a very high channel error rate involved during transmission (i.e., BER = 10^−5^), a large number of corruptions and retransmission events happen so the deferred, batch retransmissions by reliable LTP lead to much shorter file transfer times compared to the general retransmission mechanism of TCP. The performance advantage of LTP/UDP becomes larger as link delays increase. This happens because, given almost the same number of corrupted bundles and a long link delay (and, therefore, a long RTT) experienced, the deferred retransmission method of LTP leads to a shorter retransmission time for the corrupted bundles compared to the conventional immediate retransmission of TCP.

Note that the experimental result is missing for LTP/UDP transmission at a link delay of 5000 ms in Figure 3 and Table 2. According to the experimental result, when a very long delay is introduced to data transmission, LTP/UDP experiences challenges in successful transmission, especially with a high channel error rate. With an introduction of a link delay of 5000 ms or longer, LTP/UDP could not deliver any data segments successfully when a BER of 10^−5^ is involved during transmission.

**Figure 4:**
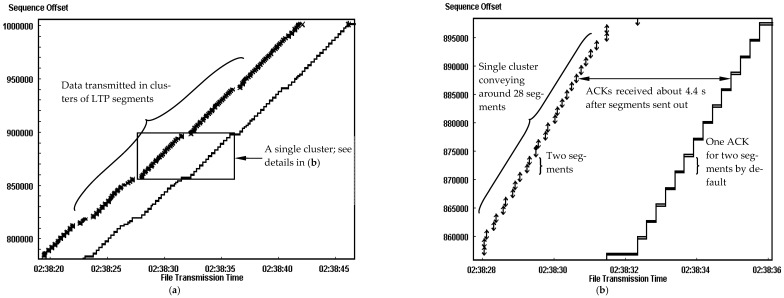
Typical traffic pattern of TCP-based DTN with a link delay of 2000 ms and error-free channel. (**a**) Segments transmission in a cluster. (**b**) Details of a single cluster.

Figure 4 provides a detailed view of the time sequence graph (TSG) [28] with a BER of 0, illustrating a typical pattern of a TCP-based DTN transmission over an error-free channel. A link delay of 2000 ms was introduced for the transmission. The file is sent in a series of segment clusters following a “send and wait for ACK” method of TCP. The method is seen because the data source node has to wait for the long-delayed ACK, which is equal to an RTT of at least 4 s. More information of the transmission can be observed from an enlarged view of a particular packet cluster (3^rd^ cluster) in Figure 4b. The entire cluster consists of 28 segments, and each ACK from the receiver acknowledges two segments by default. On average, the RTT for each segment is around 4.4 s, and it is a little longer than twice of the link delay of 2000 ms. Therefore, it can be concluded that in cislunar communication, the TCP-based DTN transmission is severely degraded by the long time spent waiting for the ACKs because of the ACK-clocked transmission, which is designed as the nature of standard TCP.

**Figure 5:**
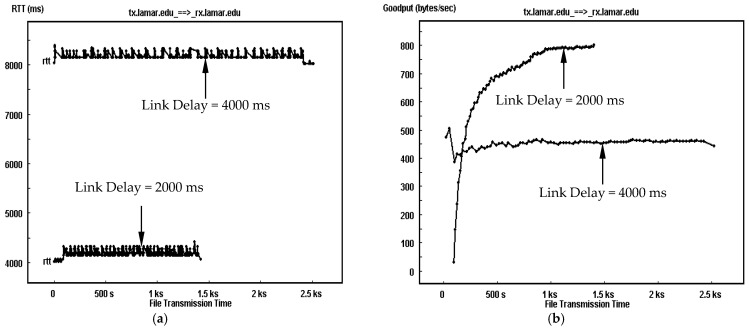
Transmission comparison of TCP-based DTN transmissions between link delay of 2000 ms and link delay of 4000 ms for a BER of 10^−5^. (**a**) Round-trip time (RTT) traces. (**b**) Goodput traces.

The performance of TCP-based transmission is aggravated by an increase in link delay when accompanied by a high channel error rate. An increment in link delay directly increases the RTT of the transmission. Figure 5 depicts a comparison of both RTT and goodput traces for TCP-based DTN with link delays of 2000 ms and 4000 ms at an error rate of 10^−5^. The experimental results show that the resulted RTT (on average) for the transmission with a one-way link delay of 4000 ms is around 8193 s, which is about twice of 4204 s with a link delay of 2000 s. This is plausible because the link delay, in comparison to other times, dominates the RTT. Corresponding to the RTT relationship between two transmissions, the averaged goodput for the transmission with a delay of 4000 ms is around 390 bytes/s, which is only a little higher than a half of 700 bytes/s for the transmission with a delay of 2000 ms, as observed in the goodput trace in Figure 5b. As a comparison, with the “keep-alive” segments excluded, their goodput is about 425 bytes/s versus 776 bytes/s. From this comparison, we see that for any two DTN transmissions with a significant difference in link delay (such as 2000 ms in our experiment), a very high channel error rate over cislunar channel only has a trivial impact on their performance difference, which is dominated by the large difference in RTT.

#### 3.2.2. Experimental Results with Link Disruption Event

**Figure 6:**
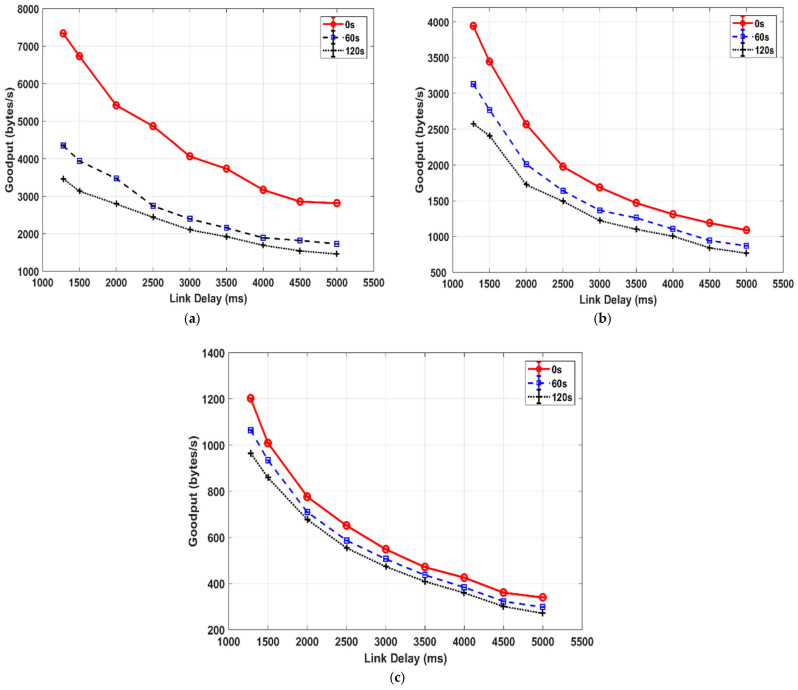
Goodput comparison of TCP-based DTN with respect to the variations of link delay and disruption length. (**a**) BER = 0. (**b**) BER = 10^−6^. (**c**) BER = 10^−5^.

Figure 6 compares the goodput for TCP-based DTN with a link disruption event involved, 60 s and 120 s, at three different BERs. Please note that the transmissions without link outages (i.e., with a disruption of 0 s) discussed in Section 3.2.1 are also included for comparison purposes, and they are represented as the one with a link disruption of 0 s. As carried out for the transmission in Figure 4, a link delay in a range of 1250 ms–5 s was configured. It is observed that given a link disruption setting (any of 0 s, 60 s or 120 s), a roughly exponential goodput decrease is observed for the linear increase in link delay for any of three settings of the channel quality.

For a given channel quality, the shorter the link delay is, the larger the goodput difference among the different settings of the link disruption is. With respect to the channel quality, the higher the channel error rate is, the smaller the goodput difference is among the different link disruption settings.

**Figure 7:**
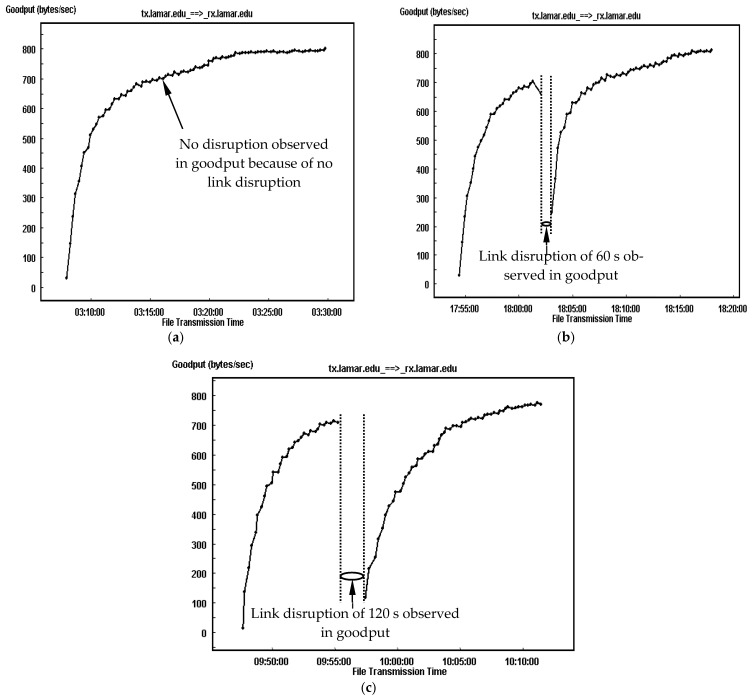
Goodput traces of TCP-based DTN for transmission with link delay of 2000 ms, a BER of 10^−5^ and three disruption events. (**a**) Disruption of 0 s. (**b**) Disruption of 60 s. (**c**) Disruption of 120 s.

To see the impact of link disruption, especially when accompanied by a high error rate, Figure 7 illustrates the goodput traces for TCP-based DTN with the disruptions of 0 s, 60 s and 120 s having a BER of 10^−5^ and a link delay of 2000 ms. For the one without any link disruption experienced (i.e., having a disruption time of 0 s), the goodput trace maintains as a single connection. In comparison, when the link disruptions of 60 s and 120 s are involved, the traces are divided into two separate segments, and the goodput has no records between two segments. This is because no data segment is transmitted when the link is disrupted. When the link is unavailable, only the link-probing SYN segments are sent, but they have no contribution to the data transmission goodput.

The experimental results show that the averaged goodput is around 700 bytes/s for the transmission without disruption. For the transmission with a link disruption of 60 s, the averaged goodput for the first segment of connection is around 556 bytes/s and is around 710 bytes/s for the second segment of connection, accomplishing an averaged goodput of around 660 bytes/s for the complete file transmission. In comparison, when the link disruption is increased to 120 s, the averaged goodput for both segments of the connections (i.e., the one prior to disruption and the one after the disruption) is around 522 bytes/s for the segment and 681 bytes/s, leading to an averaged goodput around 620 bytes/s for the complete file transmission. The goodput performance for the three complete file transmissions, 700 bytes/s, 660 bytes/s and 620 bytes/s, is reaffirmed by the different durations of the link disruption, 0 s, 60 s and 120 s, placed over the channel.

**Figure 8:**
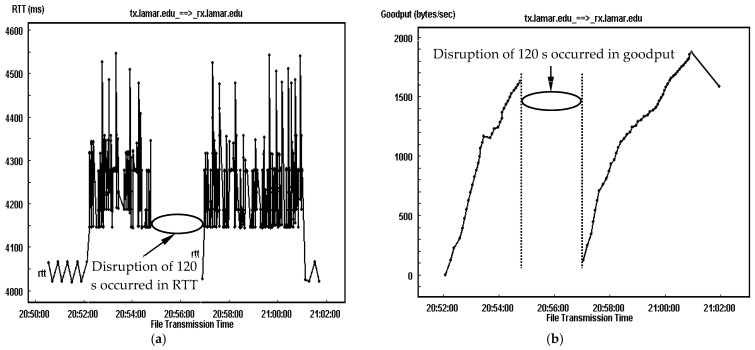
TCP-based DTN transmission have link delay of 2000 ms and a disruption event of 120 s at a BER of 10^−6^. (**a**) RTT trace. (**b**) Goodput trace.

**Figure 9:**
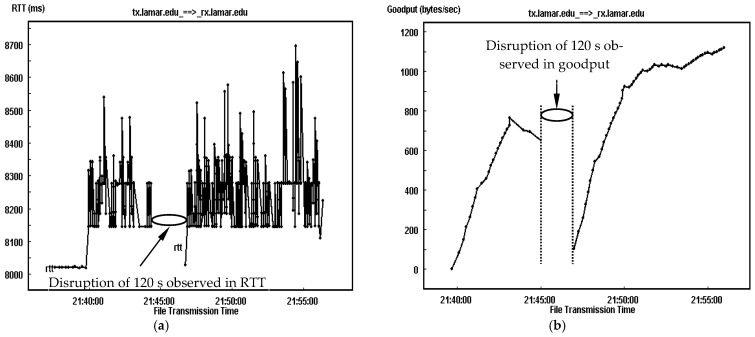
TCP-based DTN transmission having link delay of 4000 ms and a disruption event of 120 s at a BER of 10^−6^. (**a**) RTT trace. (**b**) Goodput trace.

For an illustrative understanding of the link delay impact on the transmission, Figure 8 and Figure 9 provide a comparison of the RTT trace and goodput trace for TCP-based DTN having link delays of 2000 ms and 4000 ms accompanied by a link disruption event of 120 s and an error rate of 10^−6^. Similar to the traces in Figure 7, for each link delay configuration, both RTT and goodput traces are separated as two segments and have no performance record between them as no data segments are successfully delivered during the link outage. The experimental results show that for the one with a delay of 2000 ms, the averaged RTT for both segments of connection is around 4244 ms and 4230 ms, as illustrated in Figure 8a, leading to around 4235 ms for the complete transmission. As a result, the averaged goodput is around 1087 bytes/s for the first fragment of transmission and 1568 bytes/s for the second fragment, accomplishing a goodput of around 1400 bytes/s for the complete transmission, which can be observed in Figure 8b.

In comparison, as shown in Figure 9, when the link delay is doubled, the averaged RTT and goodput for both segments are 8230 ms and 8248 ms, and 528 bytes/s and 1068 bytes/s, leading to around 8240 ms as the averaged RTT and 850 bytes/s as the averaged goodput for the complete file transmission. Clearly, the averaged RTTs for the connections with link delays of 2000 ms, 4000 ms, 4235 ms and 8240 ms are about double of their individual one-way link delay. Comparing the performance of both connections, the averaged goodput with a link delay of 4000 ms is around 550 (=1400−850) bytes/s lower than that with a delay of 2000 ms because of the significant difference in link delay.

In comparison, as shown in Figure 9, when the link delay is doubled, the averaged RTT and goodput for both segments are 8230 ms and 8248 ms, and 528 bytes/s and 1068 bytes/s, leading to around 8240 ms as the averaged RTT and 850 bytes/s as the averaged goodput for the complete file transmission. Clearly, the averaged RTT for the connections with link delays of 2000 ms and 4000 ms, and 4235 ms and 8240 ms, are about the double of their individual one-way link delay. Comparing the performance of both connections, the averaged goodput with a link delay of 4000 ms is around 550 (=1400−850) bytes/s lower than that with a delay of 2000 ms because of the significant difference in link delay.

**Figure 10:**
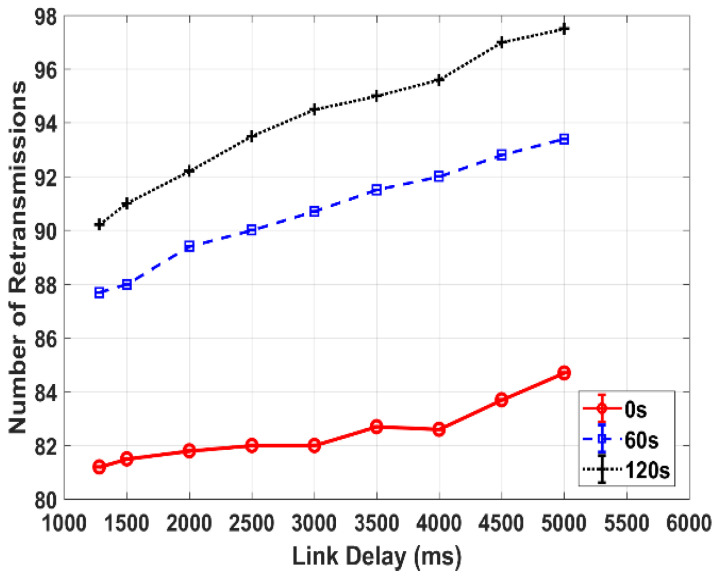
Retransmission attempts of TCP-based DTN transmissions with different link disruptions versus link delays at BER of 10^−5^.

Corresponding to the goodput decrease with respect to the increase in link delay, for a given link disruption setting, the number of retransmission events involved in data transmission increases with the link delay. This is clearly observed in Figure 10, which compares the number of retransmissions among the different link disruptions versus link delays at a BER of 10^−5^, as a sample. Similarly, at other two BER settings (i.e., 0 and 10^−6^), as the link disruption length increases, the number of retransmission events increases. At a given error rate, a transmission with a hybrid of a long delay and a very long disruption event raises the largest number of bundle retransmissions, leading to a significant decrease in goodput. In comparison, with a short link delay and short disruption experienced, the transmission leads to the least number of retransmissions events. As another observation, for the transmissions at different link delays, the number of bundle retransmissions varies drastically with a long link disruption compared to that with a short link disruption, especially at a high BER as it generally causes a large number of corrupted bundles during data transmission.

## 4. Conclusions

The experimental results show that TCP-based DTN works effectively for space-vehicle communications in cislunar domain in the presence of a link disruption event (up to 120 s in the experiment). However, a roughly exponential goodput decrease is observed with a linear increase in link delay for any of three settings of channel quality (i.e., the BERs of 0, 10^−6^, and 10^−5^). For a given channel quality, the shorter the link delay is, the larger the goodput difference among different settings of link disruption is. With respect to channel quality, the higher the bit error rate is, the smaller the goodput difference among different settings of link disruption is.

In comparison, with a given channel quality equivalent to a BER of 0 or 10^−6^, TCP-based DTN has a performance advantage over LTP/UDP. However, when experimented over a noisy channel (i.e., having a much higher BER, 10^−5^), LTP/UDP outperforms TCP-based DTN for all the experimented link delay settings in a range of 1250 ms~5 s, especially with a link delay of 2000 ms or longer. Over a noisy channel, a large number of segments are corrupted, and the effectiveness of TCP-based DTN is severely degraded by a long time in waiting for the ACKs for immediate retransmission because of the ACK-clocked nature of TCP. This leads to a significantly long file delivery time, which is aggravated by a long link delay. In comparison, it takes a much shorter time for LTP/UDP because of the deferred retransmission mechanism; therefore, a higher goodput is achieved.

The numerical results in this paper were collected based on the file transfer experiments over a PC-based experimental testbed. Even though the experiments were conducted with the real data flow that happened among different data nodes, we expect that the study results and findings on TCP-based DTN in this article can be verified over a cislunar channel during a real moon exploration mission. This verification and validation are left as the main future work when a cislunar channel becomes available.

## Figures and Tables

**Table 1:** Experimental settings.

Parameters	Settings
Protocol setting	BP/TCPCL/TCP/IP/PPP vs. BP/LTP/UDP/IP/PPP
Operating system	Red-Hat Enterprise Linux 8
Latency (one-way delay)	1280 ms~5000 s
Date rate	115,200 bit/s for each of data and ACK link
Channel quality	BERs of 0, 10^−6^ and 10^−5^
Link disruption	Without disruption With disruption (with length of 60 s and 120 s)

**Table 2:** Numerical data of goodput comparison between TCP-based DTN and BP/LTP/UDP with respect to variations of link delay and channel quality.

Link Delay (ms)	Goodput at BER = 0 (byte/s)		Goodput at BER = 10^−6^ (byte/s)		Goodput at BER = 10^−5^ (byte/s)	
	TCPCL/TCP	LTP/UDP	TCPCL/TCP	LTP/UDP	TCPCL/TCP	LTP/UDP
1280	7336.36	1911	3940.8	1883	1201.4	1675
1500	6728.15	1753	3442.3	1732	1008.1	1543
2000	5416.85	1585	2565.4	1563	775.8	1407
2500	4865.4	1345	1977.1	1343	651.7	1279
3000	4065.3	1318	1687.1	1287	548.8	1212
3500	3735.7	1149	1470.3	1144	471.4	1136
4000	3169.4	1105	1310.7	1116	425.3	1094
4500	2855.7	1010	1189.5	1008	360.7	1001
5000	2814.95	989	1090.4	911	340	-

## Data Availability

Dataset available on request from the authors.

## References

[1] De Cola T., Marchese M. (2005). Performance analysis of data transfer protocols over space communications. IEEE Trans. Aerosp. Electron. Syst..

[2] Burleigh S., Hooke A., Torgerson L., Fall K., Cerf V., Durst R., Scott K., Weiss H. (2003). Delay-tolerant networking: An approach to inter-planetary Internet. IEEE Commun. Mag..

[3] Scott K., Burleigh S. Bundle Protocol Specification, IETF Request for Comments RFC 5050, November 2007. https://www.ietf.org/rfc/rfc5050.txt.

[4] Postel J. (1981). Transmission Control Protocol―Darpa Internet Program―Protocol Specification.

[5] Postel J. (1980). User Datagram Protocol.

[6] Shao Z., Wu Q., Fan P., Cheng N., Chen W., Wang J., Letaief K.B. (2024). Semantic-Aware Spectrum Sharing in Internet of Vehicles Based on Deep Reinforcement Learning. IEEE Internet Things J..

[7] Wang X., Wu Q., Fan P., Fan Q., Zhu H., Wang J. (2024). Vehicle Selection for C-V2X Mode 4 Based Federated Edge Learning Systems. IEEE Syst. J..

[8] Zhang C., Zhang W., Wu Q., Fan P., Fan Q., Wang J., Letaief K.B. (2024). Distributed Deep Reinforcement Learning Based Gradient Quantization for Federated Learning Enabled Vehicle Edge Computing. IEEE Internet Things J..

[9] Wu Q., Wang W., Fan P., Fan Q., Zhu H., Letaief K.B. (2024). Cooperative Edge Caching Based on Elastic Federated and Multi-Agent Deep Reinforcement Learning in Next-Generation Networks. IEEE Trans. Netw. Serv. Manag..

[10] Consultative Committee for Space Data Systems (2015). Bundle Protocol Specifications.

[11] The Space Internetworking Strategy Group (SISG) (2010). Recommendations on a Strategy for Space Internetworking.

[12] Demmer M., Ott J. Delay Tolerant Networking TCP Convergence Layer Protocol. IETF DTNRG IRTF Research Group, draft-irtf-dtnrg-tcp-clayer-02.txt (Work in Progress), November 2008. https://www.ietf.org/archive/id/draft-irtf-dtnrg-tcp-clayer-02.txt.

[13] DTN Reference Implementation, October 2013 Release. https://github.com/delay-tolerant-networking/DTN2.

[14] Ramadas M., Burleigh S., Farrell S. Licklider Transmission Protocol―Specification. IETF Request for Comments RFC 5326, September 2008. https://www.ietf.org/rfc/rfc5326.txt?number=5326.

[15] Kruse H., Ostermann S. UDP Convergence Layers for the DTN Bundle and LTP Protocols. IETF DTNRG IRTF Research Group, draft-irtf-dtnrg-udp-clayer-00.txt (Work in Progress), November 2008. https://datatracker.ietf.org/doc/draft-irtf-dtnrg-udp-clayer.

[16] Wang R., Burleigh S., Parik P., Lin C.-J., Sun B. (2011). Licklider Transmission Protocol (LTP)-based DTN for cislunar communications. IEEE/ACM Trans. Netw..

[17] Lent R. (2019). Analysis of the block delivery time of the Licklider transmission protocol. IEEE Trans. Commun..

[18] Sabbagh A., Wang R., Zhao K., Bian D. (2017). Bundle Protocol over Highly Asymmetric Deep-Space Channels. IEEE Trans. Wirel. Commun..

[19] Bezirgiannidis N., Burleigh S., Tsaoussidis V. (2013). Delivery Time Estimation for Space Bundles. IEEE Trans. Aerosp. Electron. Syst..

[20] Yang G., Wang R., Sabbagh A., Zhao K., Zhang X. (2018). Modeling Optimal Retransmission Timeout Interval for Bundle Protocol. IEEE Trans. Aerosp. Electron. Syst..

[21] Sabbagh A., Wang R., Burleigh S., Zhao K. (2018). Analytical Framework for Effect of Link Disruption on Bundle Protocol in Deep-Space Communications. IEEE J. Sel. Areas Commun. Spec. Issue Adv. Satell. Commun..

[22] Zhou Y., Wang R., Yang L., Liang J., Burleigh S., Zhao K. (2022). A Study of Transmission Overhead of a Hybrid Bundle Retransmission Approach for Deep-Space Communications. IEEE Trans. Aerosp. Electron. Syst. Spec. Sect. Inf. Commun. Technol. (ICT) A New Space Vis..

[23] Wang R., Liu X., Yang L., Xi Y., Sanctis M.D., Zhao K., Yang H., Burleigh S. (2024). A Study of DTN for Reliable Data Delivery from Space Station to Ground Station. IEEE J. Sel. Areas Commun. Spec. Issue Space Commun. New Front. Near Earth Deep Space.

[24] Consultative Committee for Space Data Systems (2006). Cislunar Space Internetworking―Architecture.

[25] Simpson W. The Point-to-Point Protocol. IETF Request for Comments RFC 1661, July 1994. https://www.ietf.org/rfc/rfc1661.txt.

[26] Costello D.J., Hagenauer J., Imai H., Wicker S.B. (1998). Applications of Error-Control Coding. IEEE Trans. Inf. Theory.

[27] National Aeronautics and Space Administration (2012). Space Network Users’ Guide (SNUG).

[28] TCP Connection Analysis Tool: TCPTRACE. https://www.tcptrace.org/manual/index.html.

[29] Shepard T. (1991). TCP Packet Trace Analysis. MIT-LCS-TR-494, MIT Laboratory for Computer Science.

